# Spirituality/Religiosity: A Cultural and Psychological Resource among Sub-Saharan African Migrant Women with HIV/AIDS in Belgium

**DOI:** 10.1371/journal.pone.0159488

**Published:** 2016-07-22

**Authors:** Agnes Ebotabe Arrey, Johan Bilsen, Patrick Lacor, Reginald Deschepper

**Affiliations:** 1 Department of Public Health, Faculty of Medicine and Pharmacy, Vrije Universiteit Brussel, Brussels, Belgium; 2 Department of Internal Medicine and Infectious Diseases-AIDS Reference Center, Universitair Ziekenhuis Brussel, Brussels, Belgium; University of Stirling, UNITED KINGDOM

## Abstract

Spirituality/religion serves important roles in coping, survival and maintaining overall wellbeing within African cultures and communities, especially when diagnosed with a chronic disease like HIV/AIDS that can have a profound effect on physical and mental health. However, spirituality/religion can be problematic to some patients and cause caregiving difficulties. The objective of this paper was to examine the role of spirituality/religion as a source of strength, resilience and wellbeing among sub-Saharan African (SSA) migrant women with HIV/AIDS. A qualitative study of SSA migrant women was conducted between April 2013 and December 2014. Participants were recruited through purposive sampling and snowball techniques from AIDS Reference Centres and AIDS workshops in Belgium, if they were 18 years and older, French or English speaking, and diagnosed HIV positive more than 3 months beforehand. We conducted semi-structured interviews with patients and did observations during consultations and support groups attendances. Thematic analysis was used to analyse the data. 44 women were interviewed, of whom 42 were Christians and 2 Muslims. None reported religious/spiritual alienation, though at some point in time many had felt the need to question their relationship with God by asking “why me?” A majority reported being more spiritual/religious since being diagnosed HIV positive. Participants believed that prayer, meditation, regular church services and religious activities were the main spiritual/religious resources for achieving connectedness with God. They strongly believed in the power of God in their HIV/AIDS treatment and wellbeing. Spiritual/religious resources including prayer, meditation, church services, religious activities and believing in the power of God helped them cope with HIV/AIDS. These findings highlight the importance of spirituality in physical and mental health and wellbeing among SSA women with HIV/AIDS that should be taken into consideration in providing a caring and healthy environment.

## Introduction

Spirituality and religion can influence the way patients perceive health and disease and their interaction with other people [1–6]. Many patients are spiritual, and religious needs related to their disease can affect their mental health, and failure to meet these needs may impact their quality of life [7]. It is argued that it may be confusing to distinguish between spirituality and religion because of the ambiguous and personal meanings accorded to these concepts [4]. Spirituality is a broad concept with many perspectives and there is no consensus on a definition of this concept, only ambiguity as to how this concept is defined [8]. Spirituality is an inherent component of being human and it is subjective, intangible, and multifaceted. Spirituality and religion are often used interchangeably, but the two concepts are different. Some authors contend that spirituality involves a personal quest for meaning in life, while religion involves an organized entity with rituals and practices focusing on a higher power or God. Spirituality may be related to religion for certain individuals, but not, for example, for an atheist or yoga practitioners [8].

Similarly, some authors contend that spirituality refers to the “nearly universal human search for meaning, often involving some sense of transcendence” [9,10]. On the other hand, religion is “a set of beliefs, practices and language that characterises a community that is searching for transcendent meaning in a particular way, generally based upon belief in a deity”[10,11]. Spirituality/religion can take individual as well as collective forms. The concepts of spirituality and religiosity are not mutually exclusive and can overlap or exist separately[12]. However, prayer and meditation are often performed in solitude. Regular church attendance, religious belief or the influence of religious institutions are dwindling fast in recent years and there is also a tendency for people to believe without belonging to any religious affiliation in Western Europe and much of the developed world, irrespective of race and ethnicity [13,14]. Crockett & Voas (2006) and Voas & Crockett (2005) further assert that there is a generational decline in belief as well as religious belonging and attendance in Western Europe and much of the developed world.[13,14] On the other hand, in sub-Saharan Africa, many people still believe and belong to spiritual/religious institutions and religious plurality is common [15,16].

An increasing number of studies have examined the complexity and interdisciplinary connection between spirituality/religiosity, health and quality of life [17–19]. Recent global research and surveys have also shown that the spirituality and religious dimensions of patients’ lives need to be an integral part of patient management [20]. Spirituality/religion may differ for each person and may have a double-edged capacity that can enhance or damage health and wellbeing, especially among patients with chronic illnesses like mental disorders, cancer, diabetes, and HIV/AIDS [1,21–24].

Weathers et al 2011 give a conceptual definition of spirituality as “a way of being in the world in which a person feels a sense of connectedness to self, others, and/or a higher power of nature; a sense of meaning in life; and transcendence beyond self, everyday living and suffering” [25]. Conversely, Kaplan (2006) and Wood (1999) hold that spirituality is more than prayer, meditation, contemplation or personal reflection. It gives a sense of meaning to everyday life [26,27]. However, these scholars further assert that religion and spirituality have received increased interest in relation to serious illnesses in recent years [26,27]. Chaves (2015) conceptualized spirituality as support, relationship with the sacred, and transcendence. He also distinguished spirituality from religion, which is defined by religious affiliation, cultural affiliation and dogmas [28].

Religion and spirituality are related but distinct, as held by previous research where spirituality relates to interconnectedness with a transcendent being (spiritual perceptions) [29] and religiosity is the interpersonal and institutional engagement with a formal religious group, doctrines and traditions (frequency of religious participation) [30]. For the purpose of this study, our working definition of spirituality ‘is personal belief in God or a Higher Power, that may include individual prayer, meditation and meaning in self’ and religion is defined ‘as organisational beliefs or adherence to institutionally based belief systems or dogma’. With reference to these definitions, we aim to reduce the line between spirituality and religion, especially as some phenomena associated with spirituality are essential elements of a broad conceptualisation of religion.

The widespread use of spirituality/religion in coping with serious medical and physical conditions has also been demonstrated in literature [31,32]. A number of previous studies have examined the relationship between spirituality, religious beliefs and activities and coping with physical illness in patients with chronic health conditions like heart disease, mental disorders, renal failure, diabetes, cancer, HIV/AIDS and several other physical conditions [32–35]. It has been previously reported high spirituality/religiosity help HIV/AIDS patients cope with their disease through engaging in behavioural change, reducing anxiety and other mental problems that could arise as a result of their HIV positive status [18,19].

Sub-Saharan Africa embraces a diversity of religions that prescribe moral behaviour and teachings [36]. There are many religions (Christianity, Islam, African indigenous religions) practiced by many sub-Saharan Africans (SSA) in African and wherever they happen to be, with Christianity being the largest religion [37,38]. As evidenced in literature spirituality/religion is multifaceted and has been a major influence on health beliefs and practices [34,39–42]. It is also argued that cultural norms and values as well as religion define the health-seeking strategies of many Africans [43]. Religious belief operates at every level of the society in sub-Saharan Africa (SSA) and spirituality has become a force for wholeness, healing and inner transformation, especially for those who are disillusioned by traditional institutional religions [37]. In many sub-Saharan African (SSA) countries experiencing a breakdown of political institutions, spirituality/religion can provide liberation and solace and religious discourse can also be seen as a remedy to reordering of power [37]. Many authors posit that for a majority of SSA especially women with HIV, spirituality has become a label for meaning, values, transcendence, hope and connectedness in most African societies [19,27,44–52].

It is increasingly agreed in literature that spirituality and religion are important to many people living with HIV/AIDS [53–56]. Religious organisations in most regions of Africa are often the key providers of care and support to people living with HIV/AIDS [50,57,58]. According to the WHO 2007 report, faith-based organisations own between 30% and 70% of health infrastructures in sub-Saharan Africa and these organisations play a great role in HIV/AIDS care and treatment [59]. Religion and strong adherence to religious principles are believed to protect against HIV/AIDS transmission and other illnesses and the failure of health policy makers to understand the influence of religion in HIV/AIDS treatment and care could seriously impede efforts to improve health services [50,60,61].

Access to antiretroviral therapy (ART) has greatly increased life expectancy after a positive HIV diagnosis and care for HIV/AIDS patients has significantly changed from treatment of a terminal to a manageable, chronic medical condition [62–65]. However, the quality of life of people living with HIV/AIDS, especially women from resource-limited countries, may be greatly impacted by poor functioning, dependency on others and negative mental health conditions such as depression, guilt, anxiety, the burden of keeping a secret [66], trauma from violent conflicts and rape [67], hopelessness, fear and suicidal ideation [68–72]. In addition, negative socio-economic phenomena such as stigma and discrimination, isolation, loneliness, divorce and intimate partner violence [73–78], maternal trauma [79] and uncertainty are also challenges that may occur in life with HIV/AIDS that need more than medical care [80–86].

It has been demonstrated in literature that HIV is one of the most devastating illnesses of recent times, with profound effects on all aspects of life [87–89] and studies have shown that most persons with HIV/AIDS regard their spiritual as well as their physical health as important [90]. Most SSA women living with HIV/AIDS associate better health outcomes including self-confidence, coping, treatment adherence, longevity and coping skills to spirituality and religious involvement [91–94]. The integration of spirituality and religion in the care of patients with HIV/AIDS is important as patients face a series of challenges as a result of the diagnosis and management of the disease [45,95–99]. The World Health Organisation has stressed the need for spiritual care for patients as central and not peripheral to health [59]. The National Health Service Education for Scotland thus defines spiritual care as “that care which recognises and responds to the needs of the human spirit when faced with trauma, ill health or sadness and can include the need for meaning, for self-worth, to express oneself, for faith support, perhaps for rites or prayers or sacrament, or simply for a sensitive listener. Spiritual care begins with encouraging human contact in compassionate relationship, and moves in whatever direction need requires” [100]. Research has shown that religion and spirituality take a central place in the treatment and care of sub-Saharan Africans, in making sense of the illness and coping with HIV/AIDS [16,46,61,101–103].

The type of spirituality (negative or positive) adopted by a patient may have a critical impact on the course of the disease as reflected in previous studies [51,104,105]. It is argued that negative spirituality is when patients feel abandoned or punished by a higher power and positive spirituality means patients who firmly believe that God loves and forgives them despite their shortcomings [106]. Patients may adopt negative spiritual/religious beliefs in preference to conventional treatment that may be detrimental to health-seeking behaviours, treatment adherence, survival and quality of life [107]. For example, some HIV patients may refuse conventional treatment on the grounds that prayers and meditation will cure the virus.

Nonetheless, many HIV patients use spiritual/religious resources as enablers to stay in medical care [51,54,108]. Therefore, recognizing the type of spiritual beliefs of patients is important for holistic care and prevention by care providers [109,110]. It is also crucial for care providers to consider addressing the spiritual aspects of HIV/AIDS with SSA patients, in line with previous research that has examined the complex role of religion and spirituality in the health trajectories of patients [46,111,112]. Many studies have reported that sub-Saharan Africans with HIV/AIDS rely heavily on churches as spiritual resources, especially during the initial stage of adjusting to diagnosis, for wellbeing and longevity of life [99,113–115].

To better understand the importance of spirituality/religion as a source of strength, resilience and wellbeing among SSA migrant women with HIV/AIDS in Belgium, we need to consider the role that organised religion plays in health and illness among SSA. The dramatic transformation and salient popularity of Pentecostal and Charismatic Christianity highlights the relationship between spirituality/religion and health and wellbeing in the SSA communities, where belonging to and believing in the values of one or several Christian denominations is not uncommon [15,37,116]. The spiritual/religious diversity in SSA is mirrored among SSA migrants in a more secular Belgium and Western Europe [117–119]. With the above in mind, we have chosen to adopt the construct of spirituality/religiosity to include spiritual/religious needs identified and defined by the patients themselves, no matter how they understand it [7]. It is held by many authors that while physical assistance is given to HIV/AIDS patients, their spiritual needs should be addressed in order to provide holistic care in all stages of their illnesses [56,120,121].

## 2. Methods

This sub-study is part of a larger qualitative study on the experiences of SSA migrant women living with HIV/AIDS in Belgium. The decision to choose a qualitative approach was also based on its suitability to answer the research questions [122] and, moreover, to stress the relationship between spirituality/religion and the population studied [123]. Another reason for the choice of qualitative study is that it yields necessary information that can be helpful for decision making in healthcare and for individuals [124]. Some scholars also contend that qualitative research methods are typical approaches that are often used in anthropological and sociological research involving broadly stated questions about human experiences and realities studied through contact with people in their natural environments [125–128].

### 2.1 Study design

This is a qualitative study based on semi-structured interviews with SSA migrant women receiving HIV/AIDS treatment and care in Belgium, either identified by HIV/AIDS health care professionals from consultation lists or self-identified while attending HIV workshops as women living with HIV/AIDS. Follow-up interviews were conducted four months after the first interviews. In addition, their treating professionals were interviewed, observations were made during consultations and information from the hospital records as to their age and year of diagnosis was obtained to complement data. Where women refused to be interviewed, the HIV/AIDS healthcare providers systematically asked the patients their reasons for refusal. These reasons were communicated to the researcher who took note of the patients' reasons.

### 2.2 Study population

Participants in this study were SSA women with HIV/AIDS and HIV treatment and care providers. All the women invited were adults, aged 18 years and above, speaking French or English and receiving treatment in Belgium. Only women originating from SSA who had been diagnosed with HIV/AIDS were included in the study. Patients only recently diagnosed, within a period of less than three months, were excluded because of the great emotional impact of finding out one is HIV positive.

#### 2.2.1 HIV patients

The women were purposefully sampled from three sites:

1. *The ARC in Brussels*: HIV/AIDS treating experts purposefully selected patients meeting the criteria set for the study from the consultation list and informed them about the objectives of the study during consultations. Then they invited the patients to participate and later informed the researcher of the patient’s decision to participate or not. In the case of refusal to participate, the treating professionals communicated the reasons not to participate to the researcher who took notes of these reasons.
2. *HIV workshops in Brussels*: Self-identified HIV/AIDS patients receiving treatment and care from any of the AIDS Reference Centres were purposefully sampled through snowball techniques during HIV workshops in Belgium. During coffee breaks, the researcher approached some of the women and introduced the study to them. Those interested agreed to participate and referred the researcher to other women whom they believed would participate in the study.
3. *Support group meetings*: Two HIV support groups were selected for inclusion in the study. An HIV support group based in the AIDS Reference Centre at the Institute of Tropical Medicine in Antwerp and another HIV activist-run support group in Brussels were selected for their suitability to address the study’s aim.

#### 2.2.2 Recruitment and inclusion criteria of HIV care providers

Only healthcare providers directly involved with the treatment and care of HIV patients were invited to participate in the study. HIV experts working within the ARC of the university teaching hospital in Brussels were recruited by means of a simple oral invitation by the researcher. Sampling for the study did not aim to capture a diversity of health professionals within the entire hospital and did not claim to be representative of each ARC, as the sample was small (n = 8). The main reason for including HIV experts in the study was to access a range of views and their experiences as practitioners with a range of different professional backgrounds. It was also necessary to corroborate, verify and explore in depth some issues emanating from the women’s narratives. Prior to the start of data collection, the head of the ARC invited the researcher to the staff weekly meeting and the purpose of the study was explained to them by the researcher. The HIV experts then agreed to facilitate recruitment of participants. No informed consent form was signed by any of the HIV experts. The sample of HIV experts consisted of five physicians, a HIV therapist nurse, a psychologist and a social worker. The sample reflected the range of different professionals present in any ARC in Belgium [129]. Healthcare providers from other health services required by women with HIV/AIDS were not included in the study.

### 2.3 Data collection and study procedure

Data collection for the study was done between April 2013 and December 2014. Health care professionals identified patients that met the inclusion criteria from the consultation list, informed them about the study and invited them to participate. The treating physicians briefly explained the aim of the study to patients. Participants recruited from HIV workshops were approached and invited by the researcher to participate in the study. In both cases where they agreed to participate, they signed the informed consent forms. Interview questions were focused on their relationship with God and the role of religion/spirituality since HIV diagnosis, allowing the participants to guide the conversation to a great extent. The interviewer asked probing questions following the trend of the conversation.

All interviews were conducted in French or English and recorded digitally. As regards observations, the treating physician asked patients if the researcher could be present for observations during consultations and notes were taken based on what was observed. For example, conversations about new illness symptoms, uses of medications, patients’ opinions on current treatment and side effects were noted by the observing researcher. Additionally, certain medical examinations such as blood pressure and weight were also noted. During the data collection phase, feedback from health care professionals as to participants’ reasons for accepting or refusing to be interviewed was also noted and included in the analysis process.

### 2.4 Data analysis

All interviews were transcribed verbatim in French or English. The transcriptions and field notes from observations were then reviewed and coded in preparation for thematic analysis. Open coding was used to retrieve themes in line with the study objective and an inductive process based on grounded theory was used to identify themes as they emerged from the data. This is also known as the “bottom-up approach” [130,131]. Themes related to the topic were identified by constant comparison until saturation was reached. Two researchers (AEA and RD) read and analyzed several interviews and then compared and discussed their findings until there was consensus about the codes and their meaning [132]. In this study, the use of thematic analysis was important in the identification of new themes that recurred in the data and that could eventually produce a bigger picture leading to general explications [133]. Quotations from the data were presented with any potential identifiers removed.

### 2.5 Ethical statements

Signed informed consent was obtained from each patient in conformity with the decision of the Ethics Committees. The confidentiality of participants was respected by removing all identifying elements (country of origin, names, place of residence) from data in order not to compromise the anonymity of the study participants. Culturally sensitive words or questions related to sexual orientations and practices (homosexuality, lesbianism, or transgender) were omitted in the data collection process. Participants were free to withdraw from the study at any time. There was no financial compensation. Oral consent was also obtained from HIV care providers, who also facilitated patient selection. The Ethics Committees of the Universitair Ziekenhuis Brussel (Approval number B.U.N. 143201215911) and the Institutional Review Board (IRB) of the Institute of Tropical Medicine, Antwerp, Belgium (Approval number IRB/AB/ac/141) approved the study. The authors are prepared to submit a scanned copy of the IRB or Ethics Committee Approval at any stage of the publication.

## 3. Results

### 3.1 Characteristics of participants

This sub-study is part of a larger study that explored the challenges experienced by SSA women living with HIV/AIDS and the coping strategies they employed to cope and live well with the disease. Of the 116 invited to participate in the study, 72 refused to be interviewed and 44 accepted. The large number of women who declined to be interviewed revealed confidentiality concern, distrust of the SSA diaspora, no envisaged cure for patients, fear of stigma and discrimination, and simply not being prepared to talk about HIV/AIDS as reasons to decline active participation. None of the women who refused to participate evoked spirituality/religion as a reason for declining participation.

Table 1 shows the demographic characteristics of 44 study participants. Participants’ ages ranged from 20–67 years. They had migrated from 15 countries in sub-Saharan Africa and reasons for migrating were diverse. Thirty-two patients had been diagnosed HIV positive more than 10 years ago at the time of the interview. Thirty-eight patients found out their HIV-positive status in Belgium and 6 already knew that they were infected with HIV before leaving Africa. A majority of the women were employed or employable. All but 9 were sexually active and reported inconsistent condom use.

**Table 1:** Demographic details of study participants *(n = 44)*.

Variable	Frequency
**Age**	
** 20–29 years**	**5**
** 30–39 years**	**11**
** 40–49 years**	**15**
** 50+ years**	**13**
**Education**	
** University**	**12**
** High school**	**9**
** Secondary school**	**17**
** Primary school**	**1**
** Informal**	**1**
** Unknown**	**4**
**Mode of transmission**	
** Heterosexual**	**38**
** Homosexual**	**0**
** Service-related**	**1**
** Perinatal**	**1**
** Unknown**	**4**
**Have children**	
** Yes**	**35**
** No**	**9**
**Probable place of infection**	
** Belgium**	**5**
** Country of origin**	**39**
**Employment status**	
**Employed**	**23**
** Unemployed/jobseekers**	**13**
** Retired**	**4**
** Disability**	**4**
**Disclosure status**	
** HIV treating staff**	**44**
** Other health care professionals**	**30**
** Intimate partner only**	**25**
** Family**	**18**
** Friends**	**10**
** Children**	**9**
**Civil status**	
** Married**	**24**
** Single with partner**	**11**
** Single/widowed without partner**	**9**
**Support group adherence**	
** Members**	**19**
** Non-members**	**25**
**Reported Religion**	
** Christians**	**42**
** Muslims**	**2**

### 3.2 Spiritual/religious characteristics of participants

Forty-two women were practicing Christians and two were Muslims. About 62% of the Christians indicated they were Catholic Christians and 38% said they were Protestants/Revivalist Christians. One woman reported converting from Islam to Christianity, without giving any details as to why she changed religion, after her diagnosis. None of the women reported disclosing their HIV-positive status to anyone in the religious organizations to which they belong. Most participants indicated that spirituality was an important part of their lives. They reported attending religious services more than once a week and participated in church activities. One Christian woman indicated spiritual independence and individuality. She did not seek support by attending church regularly, but said that she did pray and meditate a lot. All the participants reported daily non-organized activities such as prayer and meditation for a greater connection with God. A few indicated fasting when it was convenient.

### 3.3 Overview of findings

A majority of the women spoke of their close personal relationship with God; most often before the question was put to them of whom they felt supported them in living with HIV/AIDS. The women discussed two common themes: strong faith and belief in God and spiritual/religious coping with illness and treatment. Many women believe that God heals in different ways and using antiretroviral therapy is a way God uses to heal them through the wisdom of the care providers. Table 2 summarises some reasons why many women combine spirituality/religiosity resources and ART in their HIV/AIDS trajectory to live a good quality life and age well. These women looked on spirituality/religiosity as a vital resource to self-regulate life with HIV/AIDS.

**Table 2:** Reasons for combining ART and God’s healing powers.

Prayer and meditation alone cannot heal HIV/AIDS
Better physical and mental health and wellbeing as a result of ART
Little or no cost in obtaining ART. Free access to ART and God
Some diseases can be treated without medications but not HIV/AIDS
Non-adherence is equivalent to not obeying God and refusing a second chance
Remaining independent to pursue goals

#### 3.3.1 Strong faith and belief in God

Reporting a relationship with and connection to God, a higher spiritual being, with a higher power was common among the women. Some women reported being connected to God or Jesus or the Holy Spirit. Connectedness with God was individual for those who believe in God. For example a woman described this connectedness that summarized participants’ beliefs about their spiritual self and God:

When I go for my daily walk, I listen to music and think of God. I believe in miracles. I never believed I was going to live to the age of 50 when I was diagnosed HIV positive 20 years ago. 10 years ago the doctors gave me up for dead when I was admitted to the emergency section of the hospital. The treating professionals were just waiting to pronounce me dead any minute. Then my pastor came and prayed for me in the hospital and left. You may say it’s psychological but no. There is a God that does things. It is my personal belief. It encouraged me to fight. Lord, let there be miracles.

(P3, age 50)

Most of the women described how their spiritual beliefs, practices or experiences changed and they discussed becoming closer to God as a result of their HIV disease. Most of the women expected a miracle to happen and that they would be cured or a cure would soon be found. A participant commented:

I am a devout Catholic. I believe in God and follow my religious practices. I was about 15 years old when I was forcibly married to an abusive 70-year-old. At 19 I was diagnosed HIV positive in Africa [Africa replaces name of country] after prolonged suffering from repeated diarrhoea, fever, loss of pregnancies and the death of my baby. One day, I escaped and ran to my parish priest who took me to the hospital because I was very sick and almost dying. My husband, a traditional medicine man, had refused to take me to the hospital. I never went back to his home. I was given refuge in a home that cared for abused girls. It was not safe for me to remain there because of the aggressiveness of my husband. The priest and his organization helped me to travel to Belgium. We have medications that we can take and survive. You must always have hope. I strongly believe in God and I always say that a miracle will happen. You must always hope and science has progressed a lot. We hope that one day we will be free of this illness.

(P1, 23 years)

For a majority of the participants, HIV diagnosis and other significant and stressful life events like genocide and the killing of very close family members, rape and intimate partner violence resulted in a reflection and increase in spirituality and perceived closeness to God. One woman perceived surviving genocide as her “second chance” to be connected to God.

I’m a Protestant with big faith (laugh)… I witnessed the massacre of my brothers and father. The diagnosis did not change my beliefs because I was already a believer. Perhaps it has helped me to endure the shock. Perhaps and I don’t doubt it. It is not because my belief and faith have increased, I say it’s because my God will do something. The disease has been diagnosed, ok, I have a powerful God. My belief added something to life, to the way I have handled the issue.

(P2, 42 years)

#### 3.3.2 Spiritual/religious coping with illness and treatment

Prayers, meditation and religious activities were the most common coping strategies reported by most participants to integrate illness and treatment into their lives. A majority of the women reported that praying and creating a connection with God helped them to accept, adjust and pursue a normal life. An illustration of this comment:

After testing for HIV, the doctor counselled me on what to expect if the results were positive…I pray a lot, a fast a lot and I believe that nobody can do anything except God. I was in great shock when the doctor told me that I’m HIV positive. I told myself I will not die if God does not want me to die, even when the doctors say you are going to die. I strongly believe in my God. The only thing I think of here is my heaven. I want to do good here and I want God to accept me for who I am. God made me not to think a lot about my illness. Nothing can happen without God’s power. I thank God that I am in Belgium and receive good care. I advise all women with HIV to take their medications as they have been asked to and also believe in God. These two things—medications and prayers—are very important.

(P13, age 41years)

Another participant commented on her relationship with God:

“I am a believer and nothing has changed concerning my relationship with God”.

(P40, age 52 years)

Some Christians with strong Pentecostal underpinnings will refuse or encourage treatment, even if it is free, based on their beliefs that only God can cure HIV/AIDS. Opposition to a strong spiritual/religious belief that only prayers and mediation are enough to cure a life-threatening illness like HIV was illustrated by a participant.

I’m in the process of divorcing after 5 years of marriage because of what I believe is the difference in integrating spirituality into my life with HIV. My husband is a very strong believer who thinks that a cure for HIV will come from God and not through medical treatment. It may be possible to be cured without medications, but I’m a realist myself. It would be stupid to think that you would be cured without taking medications. My husband wanted me to stop taking medications and only pray and fast, which I refused. He believes that God cures all illnesses. I have to take my medications even when I pray and fast.

(P23, age 31 years)

Spirituality/religiosity was commonly mentioned as the main source of psychological support among study participants.

I am a Christian and in 1982 I accepted Jesus Christ as my saviour and after the death of my husband in 1989 I decided not to go against my Christian teaching by having another man. If you have a relationship with God he will be present in the everyday challenges you encounter and not only when there is illness. If you surrender yourself to the Almighty God you will feel better because He is the one that does all. If you are with our Almighty God, Jesus Christ, life is calm and you take life as it is. There are ups and downs of life but you must trust in God…who has given the intelligence to scientists to continue searching for new and more effective HIV treatments. We must treat ourselves…despite the fact that death is the way to God.

(P21, age 65 years)

No participant reported any case of negative spiritual/religious coping and complete rejection of medical treatment in favour of prayers and meditation. However, a few cases of negative coping that privileged spiritual/religious coping through prayers, fasting and meditation over HIV therapy were reported by some HIV care providers in the case of other patients who receive treatment and care at the AIDS Reference Centre. One care provider reported:

I think another difference between an African woman and a European woman is their religion. African patients are religious. They go to church and everything. I have got a few who stopped medication because the pastor said ‘you are healed and you don’t have to take medications’. They just have to fast and not take medications. I don’t see this with European ladies.

(HIV therapist)

## 4. Discussion

This study explores spirituality/religion as a source of strength, resilience and wellbeing among sub-Saharan African (SSA) migrant women with HIV/AIDS in Belgium. Our findings have revealed that a striking proportion of participants (about 98%) indicated that spirituality/religion was a very important resource in their lives. In this study, about 96% of participants (n = 42 of 44) revealed that they were Christians and 4% were Muslims. Strikingly, this pattern mirrors HIV/AIDS prevalence rates among women in sub-Saharan Africa, a predominantly Christian region [134]. On the other hand, research has found low HIV prevalence in the growing Muslim population in SSA [135,136]. We found that most of the women in the study used spiritual/religious strategies like prayer, meditation and engaging in religious activities to buffer life stressors caused by HIV/AIDS and to adhere to ART. Many study participants reported that they prayed and meditated and always asked for divine intervention whenever they took their medications.

There is evidence from our study to suggest that although spirituality and religiousness appear to be different, they are not independent. All participants (except one who indicated only being spiritual) considered themselves both spiritual and religious in their belief in God or a Higher Power, frequent prayers, meditation, fasting, church membership and church attendance. Thus combining the spiritual/religious strategies of prayer/meditation and conventional treatment helped them cope with overall life with HIV/AIDS, consistent with some studies that described the importance of spirituality and religion in patients with chronic illness [7,137–139].

Taking medication is an individual responsibility and survival with HIV/AIDS depends on taking ART [140] and adhering to ART [141]. Research suggest that some religious beliefs and doubts about antiretroviral therapy among SSA migrant women and men may be culture specific, where faith-based healing is propagated by leaders of these faith communities. It is argued that health-related spiritual beliefs like calling on God or a higher power for protection and asking God or that power to take control of health is common among patients with life-threatening diseases like cancer, mental illness, and HIV/AIDS [107]. Patients who accept such beliefs will not overtly reject ART when offered, but most often will not adhere to treatment and will report miraculous healing. Our findings also suggest that high spirituality/religiosity may lead to voluntary or involuntary discontinuation of ART on the part of the patient due to pressure from a stronger spiritual/religious intimate partner (interview with P23, age 31) or pastor. This finding is consistent with previous research in the UK on reported deaths of three SSA women who stopped their ART on the grounds that prayers cured them of HIV/AIDS [142]. These participants, like the minority in our study, disengaged with ART because of their beliefs that were incompatible with conventional medicine. This paper also revealed that apart from the woman who identified her husband as a barrier to her adherence to treatment, most women were reluctant to identify the churches they attended and the pastors involved. Those who believed in faith-based healing described themselves simply as Christians.

Nonetheless, we do not suggest that the problem of faith-based healing affects a large number of SSA women but, if it occurs, it can have disastrous health consequences. Overall, our study revealed that spirituality/religion is very important to Christians as well as Muslim SSA women with HIV/AIDS. The most striking difference between Christian and Muslim participants was the large number of HIV/AIDS infected women among Christians as compared to Muslims. Healthcare providers should be aware of these findings in the treatment and care of HIV/AIDS infected SSA migrant women.

This study has a number of limitations. Firstly, the sample size comprised only those who agreed to be interviewed. The views of the majority (62%) on the subject could not be reported for the patients selected and invited to participate in the study refused. Therefore the findings are explicatory of spirituality/religiosity as a resource used in coping by HIV-positive sub-Saharan African migrant women in Belgium. However, sample size is less relevant in qualitative research and so the sample size is this study is adequate and we believe that saturation was achieved through the data collection methods and sample size used in this study [143–145].

Secondly, apart from the two participants who reported being Muslims, we could not report the existence of a plurality of religions among Christian participants: how many women were of the Catholic, Pentecostal/Charismatic, Protestant or other Christian denominations. In literature, dualism or plurality of religions is not uncommon among many SSA Christians. Christianity in sub-Saharan Africa denotes a tremendous diversity and this Christian sample may not be representative of different Christian denominations that exist [37,97]. Similarly, no reports were made suggesting that any participants belonged to indigenous African religions, or to other religious sects and cults. As evident from the research literature, Christian influence predominates in SSA and common practices often take the form of religious syncretism whereby people may regularly partake in Christian rituals and continue to maintain traditional beliefs and rituals [37,61,119,146–148]. However, traditional African religious norms, beliefs or rituals did not emerge in the interviews during discussions about spirituality/religiosity and illness treatment. The syncretic belief system was not revealed in our study. Further research is needed in Belgium on the impact of traditional African religious syncretism in combining different attitudes about treating HIV/AIDS.

Thirdly, potential bias in qualitative research is not uncommon because the research methods depend on the perspectives, skills, training, knowledge and experience of the researcher. There might have been bias in not including culturally-sensitive words or phrases that could denote sexual orientation of the participants, such as homosexuality, lesbianism and transgender, during the data collection process. Sexuality, sex and sexual orientations other than heterosexuality remain a subject of taboo among many sub-Saharan Africans no matter where they happen to live [149–152]. Fourthly, reports by the women may present only positive perspectives and reports of challenges may not be fully accurate, given that we could not measure spirituality/religiosity by purported attendances of religious services, organized or unorganized praying, fasting, meditation and religious activities.

It is plausible that many of these experiences may apply to other SSA migrant women living with HIV/AIDS in Belgium and that our findings could therefore foster a better understanding of the importance of spirituality/religion for SSA women with HIV/AIDS. We cannot pretend that the findings are a strict representation of the general HIV/AIDS population, but, the use of purposive sampling, triangulation and constant comparisons are pragmatic approaches used to assess validity and generalizability. The term generalisation or transferability refers to the degree to which results of qualitative research can be generalised or transferred to other situations, settings or contexts [153]. Importantly, for the purpose of this sub-study, the understanding of complex human psychosocial issues like spirituality/religion is more important than generalisability or transferability of results [154]. Furthermore, generalisation can be perceived as the “fit” between the cases “within and out” of the study, allowing representation and explication based on interpreting the original data [155]. In addition, the clear description of the sampling procedures and selection criteria provided makes generalisation achievable. Detailed information about the research sites is also provided in terms of the procedures undertaken to achieve the aims of the research. To enhance the validity of this qualitative research, different methods to collect data were used: interviews, observations and document analysis.

Despite these limitations, this study is the first to study spirituality/religiosity as a resource and its vital role among SSA migrant women with HIV/AIDS in Belgium. These findings highlight the importance of spirituality/religiosity among SSA migrant women and ways in which they express their spirituality/religiosity and how the women give context to their lives with HIV/AIDS. The study further provides insights into the perceived role of spirituality/religiosity in illness, health and healing, consistent with previous research [45,95,156]. Nonetheless, integrating spirituality and religion in health and professional training has met with difficulties because most healthcare professionals lack the training on how to deal with the spiritual/religious dimension of health and disease [157]. Some authors further argue that spirituality/religion are incompatible with the concept of health [158].

There is an entire body of literature supporting the benefits of spiritual/religious coping in persons with chronic illnesses like HIV/AIDS [34,54,159]. Our study findings further confirm the very important role spirituality/religiosity play in the management of life stressors [160] and the overall physical and mental health and wellbeing of HIV-infected SSA migrant women, consistent with previous research [6,112]. This study also revealed that strong faith and belief in God can help the HIV-infected women cope with their illness and stay on medications as they strongly believe that their treatment trajectory is determined by God. Most participants indicated that if God had wanted them to die of HIV/AIDS, he would not have made it possible for them to be in Belgium, where they get free treatment and care. Access to free antiretroviral therapy (ART) has made living with HIV/AIDS for these women less disastrous, especially for those who can tolerate the therapy. This assertion came especially from women originating from SSA countries where sexual violence had been used as a weapon of war and millions of people had died in internal violent conflicts [161,162]. Their spirituality/religiosity permitted them to step back and look at their lives from different perspectives. They perceived that God had forgiven them and had given them a second chance, which they must not abuse. These women revealed that they were happy to be alive, though living with a terrible disease that sometimes causes shame [163–165].

Spirituality/religion as an incentive towards treatment adherence is highlighted in our study, as participants developed a positive self-image, self-care, positive health behaviours and self-esteem. Furthermore, the participants reported that ART and strong faith and belief in God had reduced their worries of imminent death and coping with other HIV/AIDS-related illnesses that they are confronted with. The participants believed that they do their part, that is to take their medications, and God will do his part, which is to make them feel well and happy. This combination of ART and God’s hands in the healing trajectory of women with HIV/AIDS has been reported in previous studies [166]. This finding provides support to previous research holding that strong faith and belief in God enable HIV positive SSA migrant women to worry less about dying [167]. We found that participants saw spirituality/religion as connection to God, and not to the religious communities to which they belong, for fear of stigmatization and discrimination. Interestingly, not disclosing HIV positive status to any religious leader indicated their connectedness only to God or a higher being.

Future research should focus on HIV-infected women who abandon HIV care for religious/spiritual reasons and later return to restart ART when they are in a serious and critical condition. There is a need for an in-depth understanding of the major reasons for this and the process they undergo in refusing or restarting medical care.

## 5. Conclusions and Relevance to Clinical Practice

The findings of this study illustrate the necessity for researchers, clinicians and policy makers to recognise the many meanings and relevance attributed to spirituality/religiosity by SSA migrant women in the Belgian context. This study demonstrates that when SSA migrant women become ill with HIV/AIDS, they tend to heavily rely on spirituality, religious beliefs and practices in order to relieve stress, maintain hope and a sense of control, meaning and purpose in life, as has been found in previous studies of patients with chronic illnesses [45,98,168,169]. Our study concludes that spirituality/religiosity among SSA migrant women can help them cope better with the experience of living with HIV/AIDS. However, there is also the possibility of high spirituality/religiosity as an incentive or a barrier to treatment adherence among SSA women living with HIV/AIDS. Despite the diversity of faiths, healthcare providers should learn details of specific religious perspectives by talking and listening to SSA women with HIV/AIDS as proposed in other studies [34,170–172]. Patients are quite aware of the power of medical science and doctors, but generally patients are influenced by their communities, especially when these communities are formed around cultural religious practices [56,173].

Several studies have shown that the doctor-patient relationship is more than the provider-consumer model, for healthcare providers not only supply services desired by the healthcare consumers but engage patients in negotiations that persuade and push them to adhere to treatment [105,174]. When spirituality/religiosity becomes a hindrance to therapy adherence, care providers should be aware of and acknowledge the possible co-existence of spirituality/religiosity and ART adherence among many SSA women. Care providers should use examples of patients who combine spirituality/religiosity and conventional treatment to persuade and counsel “divine-healing” oriented patients to remain in medical care and continue treatment. From this study, spiritual/religious involvements enable SSA women with HIV/AIDS to cope better and experience mental/psychological wellbeing despite their negative health outcomes if antiretroviral therapy is disengaged.

## 6. Recommendations

Patients and health caregivers can benefit from organized spiritual/religious interventions despite sensitive cultural differences. Healthcare providers should improve their practical approaches with HIV patients by considering ways to assess and identify how spirituality serves as a basis for giving meaning to the patient’s experience of HIV. Religious and spiritual coping should be assessed and encouraged, without pressurising patients, early in treatment in order to help patients who express the use of religious/spiritual resources to cope. Spiritual/religious coping techniques and appropriate interventions, including discussing religious/spiritual beliefs, referral to pastoral and humanist counsellors and psychotherapy to address religion/spirituality, should be incorporated into treatment plans.
